# Triptolide improves systolic function and myocardial energy metabolism of diabetic cardiomyopathy in streptozotocin-induced diabetic rats

**DOI:** 10.1186/s12872-015-0030-4

**Published:** 2015-05-13

**Authors:** Zhongshu Liang, Sunnar Leo, Helin Wen, Mao Ouyang, Weihong Jiang, Kan Yang

**Affiliations:** Department of Cardiology, Third Xiangya Hospital, Central South University, Changsha, Hunan, 410013 People’s Republic China

**Keywords:** Triptolide, Diabetic cardiomyopathy, ^31^P NMR spectroscopy, Cardiac energy metabolism, MAPK

## Abstract

**Background:**

Triptolide treatment leads to an improvement in Diabetic Cardiomyopathy (DCM) in streptozotocin-induced diabetic rat model. DCM is characterized by abnormal cardiac energy metabolism. We hypothesized that triptolide ameliorated cardiac metabolic abnormalities in DCM. We proposed ^31^P nuclear magnetic resonance (^31^P NMR) spectrometry method for assessing cardiac energy metabolism in vivo and evaluating the effect of triptolide treatment in DCM rats.

**Methods:**

Six weeks triptolide treatment was conducted on streptozotocin-induced diabetic rats with dose of 100, 200 or 400 μg/kg/day respectively. Sex- and age-matched non-diabetic rats were used as control group. Cardiac chamber dimension and function were determined with echocardiography. Whole heart preparations were perfused with Krebs–Henseleit buffer and ^31^P NMR spectroscopy was performed. Cardiac p38 Mitogen Activating Protein Kinase (MAPK) was measured using real time PCR and western blot analysis.

**Results:**

In diabetic rats, cardiac mass index was significantly higher, where as cardiac EF was lower than control group. ^31^P NMR spectroscopy showed that ATP and pCr concentrations in diabetic groups were also remarkably lower than control group. Compared to non-treated diabetic rats, triptolide-treated diabetic groups showed remarkable lower cardiac mass index and higher EF, ATP, pCr concentrations, and P38 MAPK expressions. Best improvement was seen in group treated with Triptolide with dose 200 μg/kg/day.

**Conclusions:**

^31^P NMR spectroscopy enables assessment of cardiac energy metabolism in whole heart preparations. It detects energy metabolic abnormalities in DCM hearts. Triptolide therapy improves cardiac function and increases cardiac energy metabolism at least partly through upregulation of MAPK signaling transduction.

## Background

Diabetic cardiomyopathy (DCM) is one of the most common diabetes-associated complications encountered in the clinical practice [1]. DCM has been known to impair the function of the cardiac muscle and has been associated with high morbidity and mortality rate [2–5]. DCM occurred independently of coronary artery disease and hypertension [2, 6, 7].

Numerous studies on DCM, either on animal or molecular study, have found that myocardial abnormal glucose utilization and the shift toward fatty acid oxidation are the major pathophysiological alterations that may lead to diabetes mellitus (DM)-associated myocardial remodeling and heart failure [2, 4, 5, 7–11].

In diabetic rat model, we previously demonstrated the significant improvement on myocardial remodeling following triptolide treatment [12]. The inhibition of inflammation process by triptolide was evident [12–14]. However, whether triptolide ameliorates cardiac metabolic abnormalities remains unclear [15]. Results from limited studies has suggested that Mitogen Activated Protein Kinases (MAPK) may play an important role as MAPK has been known as an important factor that interacts with mitochondria in the production of ATP [16]. Evidence from previous study revealed that Triptolide treatment could strongly activate MAPK signal transduction pathways in cells. These findings can be really intriguing as one may speculate that Triptolide treatment may improve cardiac energy metabolism by upregulating MAPK signal transduction [17]. Therefore, we sought the alteration of MAPK signaling transduction in rat model following the induction of DCM and following triptolide treatment. Recently, nuclear magnetic resonance (NMR) spectroscopy has been applied extensively in biomedical field [18, 19]. As a non-invasive diagnostic method, NMR spectroscopy has advantage as it allows determination on dynamic changes of specific metabolites in intact organs or tissues [20, 21], such as phosphocreatine (pCr), adenosine triphosphate (ATP), inorganic phosphate (Pi), and intracellular pH (pHi) [22–24]. In addition, NMR spectroscopy allows real-time observations on physiological function and energy metabolism of certain organ (e.g. heart) in near-physiological condition [20].

In this study, ^31^Phosphorus NMR (^31^P NMR) spectroscopy was used to evaluate the effect of triptolide treatment on the cardiac energy metabolism in DCM rat model. To minimize interference [25], we decided to perform the measurement *in vitro*.

## Methods

### Animal model and treatment

The protocols used in this study were approved by the Committee of Animal Care and Use of Central South University. Eight weeks old male Sprague–Dawley (SD) rats (Animal Center of Central South University, China) were included in the study. Animals were placed in laminar flow cages on a 12 h dark and 12 h light cycle and were fed with standard chow and tap water ad libitum. DM was induced by injecting streptozocin (STZ, 70 mg/kg, dissolved in 0.1 M sodium citrate buffer, pH 4.5; Sigma, USA) intra-peritoneally after overnight fasting. Random blood glucose levels were measured at 3 days and 1 week following the injection using One Touch Sure Step glucometer (LifeScan, USA). Tail vein bloods were used and only rats with blood glucose level > 16.7 mmol/l in both time points were finally used. All the diabetic animals were randomized into four groups (n = 12 each): three diabetic groups treated with triptolide (100, 200, or 400 μg/kg/day respectively) and one diabetic group treated with vehicle. 12 sex- and age-matched non-diabetic rats served as control group. In addition, to assess the side effects of triptolide treatment, 12 sex- and age-matched non-diabetic SD rats (intraperitoneal injection of sodium citrate buffer) were treated with triptolide 400 μg/kg/day. After dissolved in dimethylsulfoxide (DMSO), Triptolide (Chinese National Institute for the Control of Pharmaceutical and Biological Products, China) was administered via gastric irrigation once daily for 6 weeks. At the end of this study, cardiac function was assessed and animals were sacrificed. The hearts were quickly extirpated and subjected to biochemical analysis [12].

### Cardiac function measurement

Echocardiography was performed using GE Vivid 7 (General Electric, USA) ultrasound system with a 10-MHz transducer. Prior to the examination [12, 26], rats were anesthetized with pentobarbital (50 mg/kg intraperitoneally) and fixed in the supine position. LV end-diastolic dimension (LVEDD) as well as LV end-systolic dimension (LVESD) were measured on the parasternal long axis view and were indexed to body weight. LV ejection fraction (LVEF) was also calculated. All measurements were performed in triplicate by an experienced investigator who was blinded to the study and the results were expressed as the average of obtained value.

### ^13^P NMR spectroscopy

^31^P NMR spectroscopy was performed in a whole heart as previously described [27]. Briefly, the heart was perfused with modified Krebs–Henseleit buffer (11 mmol/L glucose, 4.5 mmol/l pyruvate, and 0.5 mmol/l lactate, no phosphate) at constant flow rate (15 ml/min) and pressure (100 mmHg) [28]. The heart was put into 25 mm NMR tube and subjected to 400 MHz 9.4 T vertical wide bore superconducting magnet (BrukerBioSpec 9.4 T Animal MRI System, Switzerland). The temperature of the heart was kept at 37 °C during the procedure. Peak resolution was enhanced by shimming the proton signal to a line width between 20 and 35Hz. Using a spectrometer (Varian, Palo Alto, CA), consecutive 4 minutes of the spectra were acquired at 161.92 MHz.

Using a computer program (NMR1, Tripos, St. Louis, MO), the areas of the spectral peaks were fitted to sum of Lorentzian and Gaussian line shapes. After adjustment for spectral saturation, absolute ^31^P concentrations were calculated by adding Atriptolide 10.6 mmol/l to the initial h-ATP peak area and calculating ATP and PCr peak areas relative to this area. pH was estimated from the chemical shift of the inorganic phosphate (P_i_) peak (δ_Pi_) relative to that of the PCr peak.

Solutions and ChemicalsA phosphate-free Tyrode solution was used in heart NMR which contained 136.3 mM NaCl, 5.4 mM KCl, 1.0 mM MgCl2, 0.9 mM CaCl2, 10.0 mM glucose, and 5.0 mM HEPES [16, 25]. The solution was pre-warmed to 40 °C and oxygenated with 100 % Oxygen. For a Na-free solution, Na + and Ca2+ were replaced with *N*-methyl-Dglucamine on an equimolar basis as follows: 137.2 mM *N*-methyl-D-glucamine, 5.4 mM KCl, 1.0 mM MgCl2, 10.0 mM glucose, and 5.0 mM HEPES. Phosphate-free KH solution contained 118 mM NaCl, 5.9 mM KCl, 2.5 mM CaCl_2_, 1.2 mM MgSO4, 25 mM NaHCO3, 12 mM glucose, and 0.5 mM Na_2_EDTA. The KH solution was oxygenated with 95 % O_2_-5 % CO_2._

### Real time polymerase chain reaction

Cardiac RNAs were extracted using TRIzol Reagent (Invitrogen, CA, USA) according to the company’s protocol. After first strand cDNA synthesis, SYBR Green Real Time-PCR was performed using SBYR Premix Ex Taq (Takara Bio Inc., Japan). The sequences of the primers were p38: Forward Primer 5'-TCCAAGGGCTACACCAAATC-3', Reverse Primer 5'-TGTTCCAGGTAAGGGTGAGC-3'; β-actin Forward Primer 5'-GAGAGGGAAATCGTGCGTGAC-3',Reverse Primer 5'-CATCTGCTGGAAGGTGGACA-3'. The specificity of each PCR product was validated by the melting curve analysis. The expressions of mRNA were determined by constructing the differences between the cycle thresholds (Ct): ∆Ct = Ct gene of interest − Ct housekeeping gene. The conversion of ∆Ct to relative gene expression is fold induction of 2^−∆Ct^.

### Western blot analysis

Cardiac proteins were extracted using radio immune precipitation assay buffer. Protein concentration was determined using BCA Protein Assay Kit (CW0014, Beijing CoWin Bioscience Co. Ltd., China) according to the manufacturer’s protocol. Denaturated proteins were loaded into every single well and were separated by SDS-PAGE gel. Gels were transferred to an Immobilon-P membrane at 290 mA. The antibodies for phosphop38 MAPK and b-actin were purchased from Cell Signaling Technology (Beverly, MA). The expression of these proteins in the membrane was detected using an enhanced chemiluminescence kit (Western Bright, Advansta Co., U.S.A.).

### Statistical analysis

All data were expressed as mean ± SD and compared by one way ANOVA with Tukey’s post-hoc test using SPSS 16.0 (SPSS, Inc, Chicago, IL). The correlation between variables was calculated using linear regression analysis. Statistical significance was defined as p < 0.05.

## Results

All mice treated with Streptozocin developed hyperglycemia. During the whole study, no evidence of ketoacidosis was observed in all diabetic mice. No significant change of blood pressure was observed in all animals. Compared to non-diabetic groups, all diabetic groups showed higher cardiac mass index (all p < 0.05). The increased mass indexes were mainly due to the significantly smaller body weight of the animals in these groups. Comparison of blood glucose level and cardiac mass index between control group and non-diabetic + Triptolide group showed no significant differences (Table 1).

**Table 1:** General data

	Control	TP	DM	DM + TP, L	DM + TP, M	DM + TP, H
Glucose (mmol/l)	6.7 ± 2.0	5.8 ± 1.5	34.3 ± 2.7*	33.3 ± 3.7	31.2 ± 3.3*	33.4 ± 2.9*
BW (g)	462.0 ± 21.5	470.0 ± 21.2	213.3 ± 20.1*	236.5 ± 38.4*	234.6 ± 33.1*	225.7 ± 30.3*
HW (mg)	1190.3 ± 15.3	1210.2 ± 13.4	756.5 ± 12.6*	763.5 ± 14.8*	779.4 ± 15.2*	736.5 ± 14.1*
HW/BW (mg/g)	2.37 ± 0.33	2.40 ± 0.31	3.92 ± 0.48*	3.40 ± 0.46*	3.10 ± 0.46*^#^	3.01 ± 0.54*^#^

BW body weight, HW heart weight, TP,L low-dose triptolide (100 μg/kg/day), TP,M medium-dose triptolide (200 μg/kg/day), TP,H high-dose triptolide (400 μg/kg/day). ^*^P < 0.05 versus Control; ^#^P <0.05 versus DM

### Cardiac performance

**Fig. 1:**
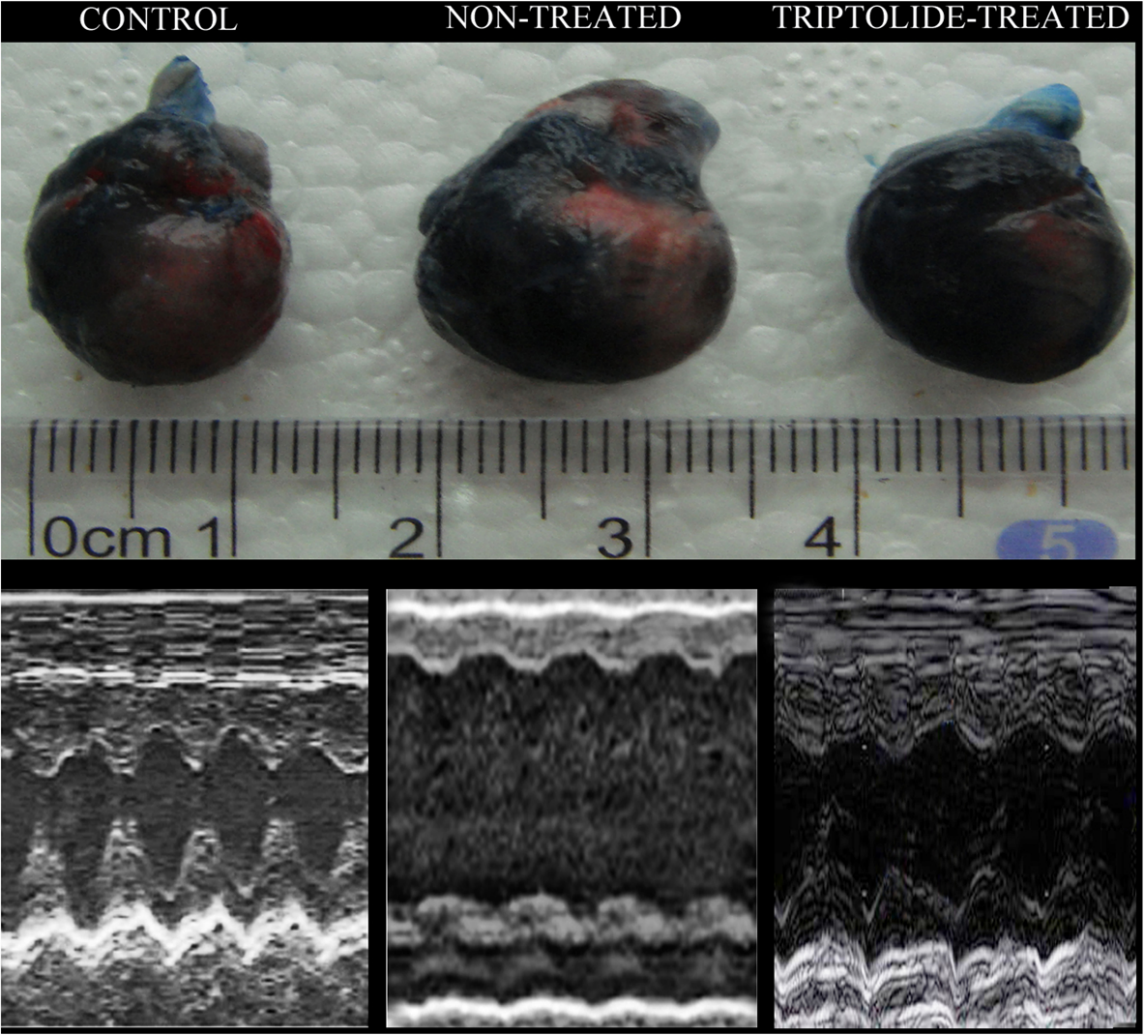
Comparison of cardiac gross anatomy and systolic function between groups

When indexed to the body weight, both LVEDD and LVESD indexes were significantly higher in diabetic than in non-diabetic rats. LV systolic function, as evidenced by LVEF, was significantly higher in groups treated with Triptolide when compared to non-treated group. Moreover, FS in triptolide-treatment diabetic groups also showed the upward trend compared with the untreated diabetic group, but the difference did not reach statistical significance (Table 2). The comparison of cardiac size and function between groups is displayed on Fig. 1.

**Table 2:** Echocardiographic parameters

	Control	TP	DM	DM + TP, L	DM + TP, M	DM + TP, H
LVEDD, mm	6.4 ± 0.6	6.5 ± 0.7	5.9 ± 0.5	6.0 ± 0.6	5.6 ± 0.6	5.4 ± 0.8
LVEDD index, um/g	13.8 ± 1.6	13.7 ± 2.1	23.6 ± 3.0*	21.1 ± 1.9*	20.2 ± 1.5*^#^	19.0 ± 1.8*^#^
LVESD, mm	3.9 ± 0.4	3.8 ± 0.7	3.7 ± 0.6	3.8 ± 0.8	3.3 ± 0.5	3.2 ± 0.8
LVESD index, um/g	8.4 ± 0.8	8.3 ± 0.7	15.8 ± 1.9*	13.5 ± 1.7*	12.9 ± 1.3*^#^	12.5 ± 1.6*^#^
LVEF,%	76.4 ± 8.2	78.2 ± 6.3	66.6 ± 6.5*	72.8 ± 5.5	75.0 ± 5.8^#^	74.6 ± 6.4^#^
FS,%	44.7 ± 4.3	43.7 ± 5.1	35.8 ± 3.6*	38.9 ± 4.1	41.3 ± 4.9	42.4 ± 4.6

LVEDD left ventricular end-diastolic dimension, LVESD left ventricular end-systolic dimension, LVEF left ventricular ejection fraction, FS fractional shortening. TP,L low-dose triptolide (100 μg/kg/day), TP, M medium-dose triptolide (200 μg/kg/day); TP, H high-dose triptolide (400 μg/kg/day). *P < 0.05 versus Control; ^#^P < 0.05versus DM

**Fig. 2:**
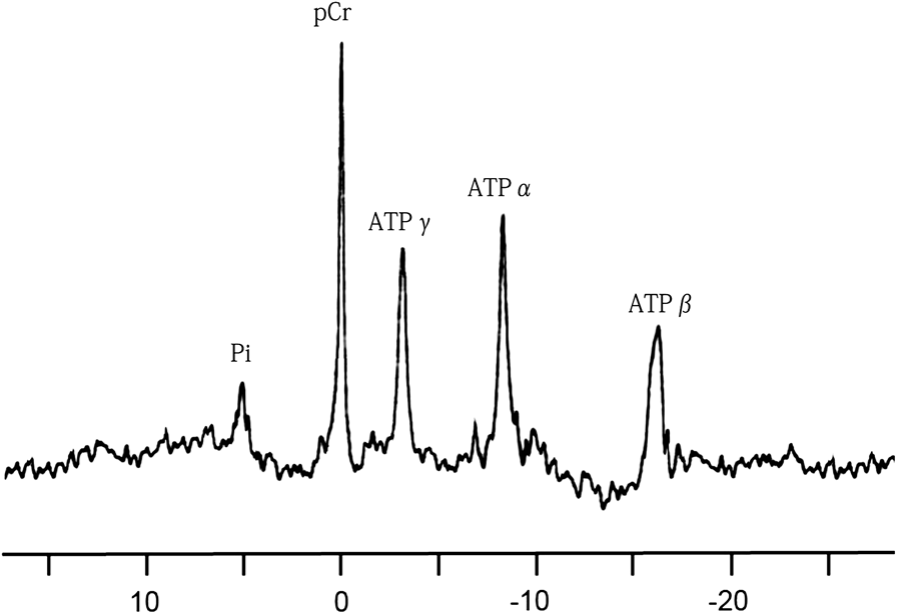
Representative image of ^31^P NMR spectroscopy

^31^P NMR spectroscopy demonstrated that the values of pHi, ATP and pCr were significantly lower in the untreated diabetic rats as compared to non-diabetic rats (Table 3). A trend of increasing pHi, ATP, and pCr was observed following Triptolide treatment. Representative image of ^31^P NMR spectroscopy is displayed in Fig. 2.

**Table 3:** pHi values and concentrations of ATP and pCr in whole heart preparations treated at varying doses of triptolide

	Control	TP	DM	DM + TP, L	DM + TP, M	DM + TP, H
pHi	7.26 ± 0.12	7.24 ± 0.14	7.20 ± 0.12*	7.22 ± 0.12	7.24 ± 0.12#	7.23 ± 0.12
ATP (mmol/L)	0.17 ± 0.03	0.18 ± 0.01	0.07 ± 0.01*	0.10 ± 0.02*^#^	0.13 ± 0.02*^#^	0.14 ± 0.01*^#^
pCr (mmol/L)	21.3 ± 1.3	21.5 ± 2.8	13.7 ± 1.3*	16.6 ± 1.7*^#^	18.8 ± 2.3*^#^	18.9 ± 2.2*^#^

TP,L low-dose triptolide (100 μg/kg/day), TP,M medium-dose triptolide (200 μg/kg/day), TP,H high-dose triptolide (400 μg/kg/day). ^*^P < 0.05 versus Control; ^#^P < 0.05 versus DM

### MAPK Signaling pathway

**Fig 3:**
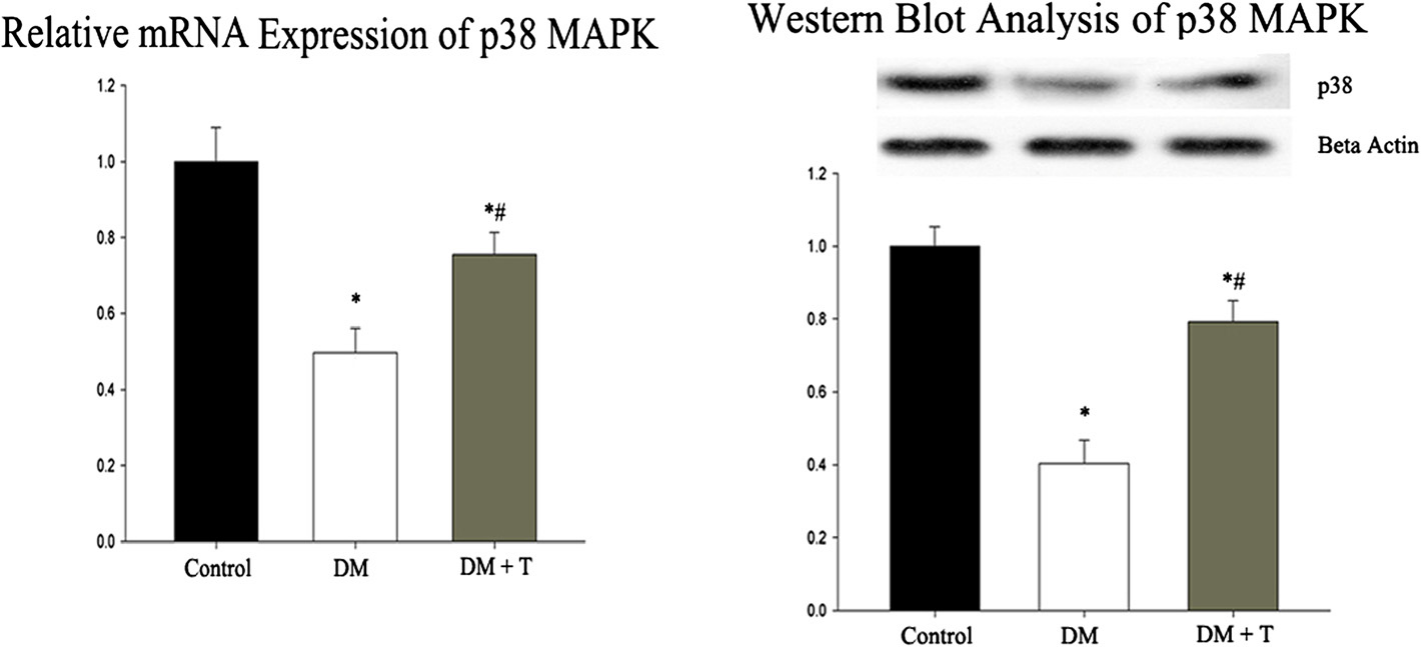
Relative mRNA and protein expression of cardiac p38 MAPK in different groups

There was a two-fold decrease in p38 mRNA expression in diabetic rats when compared with control. p38 mRNA expression was found significantly higher in diabetic rats treated with Triptolide. Consistently, on Western blot analysis, both control and Triptolide-treated groups showed stronger bands for p38 protein expression than those of diabetic group (all p < 0.05, Table 4, Fig. 3).

**Table 4:** p38 mRNA and protein expression (mean ± SD)

Parameter	Group		
	Control	DM	DM + TP
VEGF mRNA (2^-∆Ct^)	0.116 ± 0.08	0.060 ± 0.03*	0.086 ± 0.03*^#^
PKG-1 protein expression (OD)	0.912 ± 0.18	0.413 ± 0.15*	0.704 ± 0.13*^#^

∆Ct, Ct gene of interest − Ct beta actin; OD, optical density as indexed to beta actin; DM, Diabetes Mellitus; TP, Triptolide. ^*^P < 0.05 versus Control; ^#^P < 0.05 versus DM

### Electron microscope

**Fig. 4:**
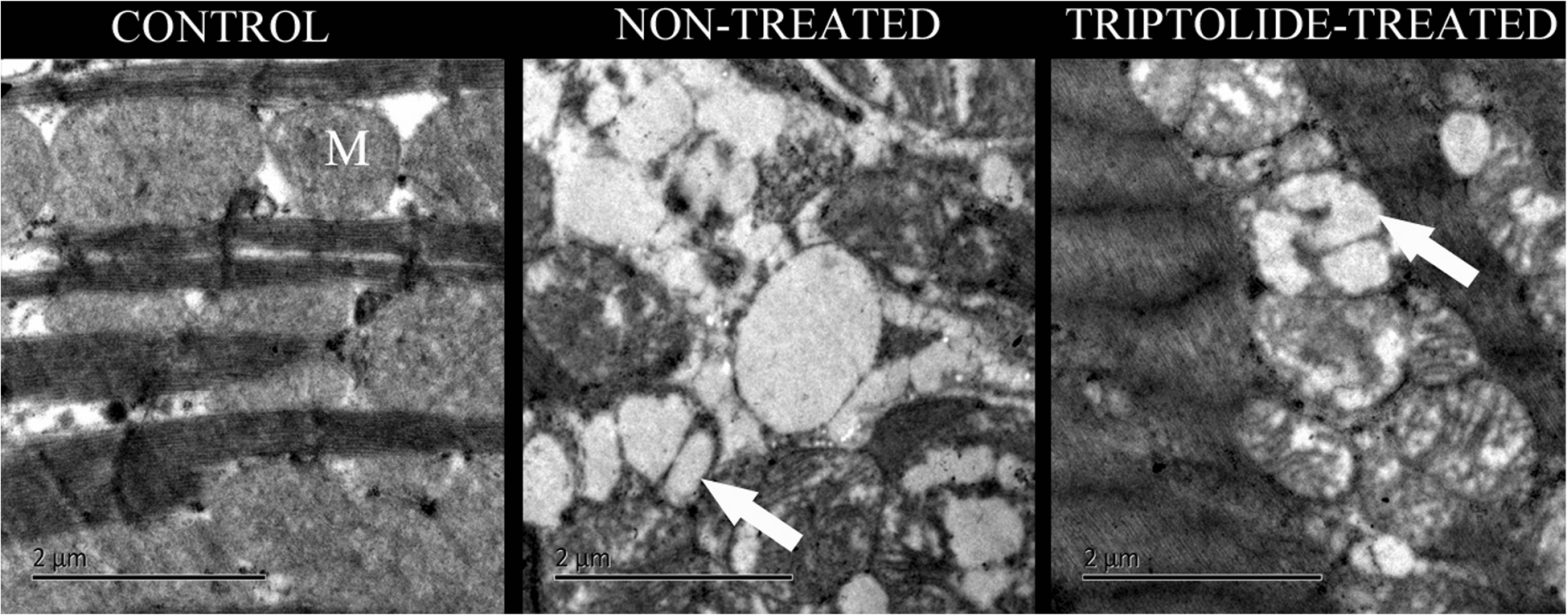
Electron microscopic analysis of cardiomyocyte. M, Normal mitochondria; Arrow, Mitochondria with vacuolar degeneration

In diabetic rats, myocardial filaments were not intact. Mitochondria were disorganized with obvious vacuolar degeneration (Fig. 4). In Triptolide-treated diabetic group, myocardial filaments were relatively more intact with remarkable lesser vacuolar degeneration of mitochondria. In contrast, no evidence of vacuolar degeneration of mitochondria was found in control group.

### Correlation analysis

**Fig. 5:**
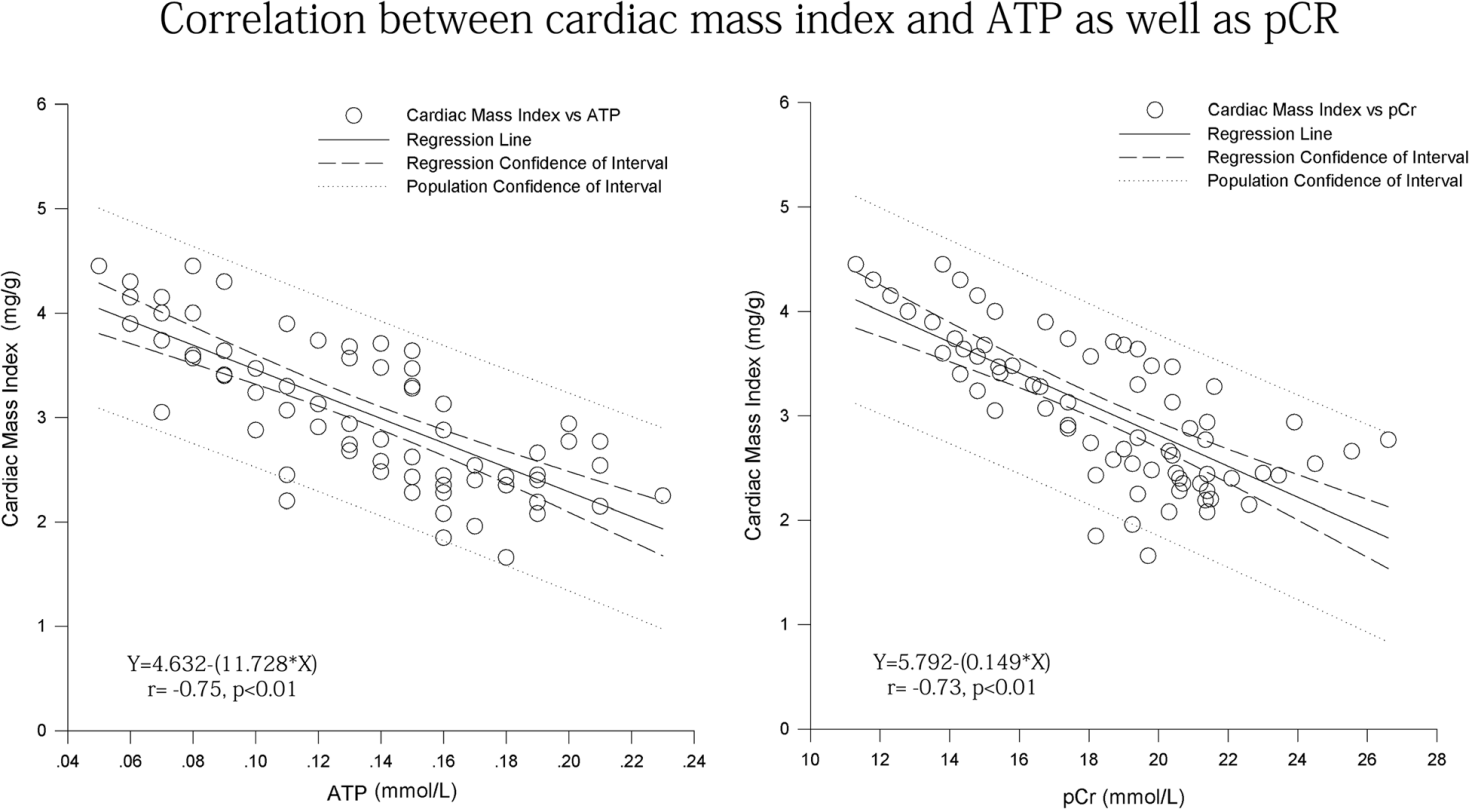
Linear regression analysis between cardiac mass index and ATP as well as pCr

The correlation between cardiac mass index and ATP as well as pCr was examined by linear regression analysis. Cardiac mass index was negatively correlated with ATP and pCr (r = −0.75 and r = −0.73 respectively, all p < 0.01, Fig. 5).

## Discussion

Diabetes is the most common endocrine disease encountered in the clinical practice [29]. DM causes series of metabolic disorders, such as glucose, lipid and protein metabolism. Long term diabetes can lead to multi-system of organ damage [23]. Recent studies have associated mitochondrial dysfunction and DM [8, 29–31]. Mitochondria have been known asthe sites of energy metabolism. Mitochondrial dysfunction is one of the characteristics of DM and has occurred even in the early stage of the disease [5]. Increasing evidences have supported that mitochondrial energy metabolism dysfunction played a crucial role in the pathogenesis of DM [11, 26, 32]. Hence, we performed ^31^P NMR spectroscopy in order to evaluate the energy metabolism in DCM rats. Secondarily, we sought to evaluate if Triptolide could improve myocardial energy metabolism.

Electron microscope is the common method used for evaluating the amount and structure of mitochondria. Cardiac muscle biopsy allows the measurement of ATP production [20–22]. However, all of these methods are invasive and can only be performed in vitro. In daily clinical setting, such kind of method is often found impractical. Therefore, a non invasive and reliable technique is needed. Japanese researchers found that both ^31^P NMR detection and cardiac biopsy showed the similar result of myocardial energy metabolism [27]. This finding makes phosphorus NMR spectroscopy is more favorable providing that it is a non-invasive technique and can be performed in vivo. Since the chemical shift peak of pCr is stable and is not influenced by internal environment, it is often used as a standard to determine the remnant compounds of chemical shift such PME, Pi, PDE and ATP [22, 24]. Energy metabolism can be assessed by quantifying area calculation under the peak of each remnant compound, in which ATP is a direct energy supplier whereas pCr is energy storage [22, 24, 25]. When muscle contracts, pCr transmits high energy phosphate bond to ATP for energy supply, conversely, when muscle relaxes, the reaction of oxidative phosphorylation in mitochondrial membrane generates ATP and then the energy is transferred to pCr for storage [25, 27, 33]. Furthermore, phosphorus spectrum enables to calculate intracellular pH, assesses the degree of anaerobic glycolysis, and evaluates the efficiency of aerobic metabolism in cells [8, 34], which become an important indicator for assessment of mitochondrial function [25, 28].

Our previous study has demonstrated that cardiac systolic function was impaired in diabetic rats and the cardiac index increased significantly [12]. Consistently, we observed the hemodynamic improvement following triptolide treatment. However, the exact mechanism involved in the hemodynamic improvement remains speculative. We believe the hemodynamic improvement following the treatment may be due to the anti-fibrotic property of Triptolide. Based on our previous findings, pro-fibrotic action of NF Kappa B in STZ mice were effectively suppressed by Triptolide [12]. Moreover, Triptolide treatment led to the inhibition of inflammatory cytokines such as Tumor Necrosis Alpha and Interleukin-1 which eventually attenuated the cardiac inflammation. We speculate that the Triptolide anti-fibrotic effect may lead to the improvement in ventricular remodeling and myocardial contraction. This, in turn, will ultimately improve the hemodynamic status of the failing hearts.

In this current study, we demonstrated that high-energy phosphate metabolism of the whole heart can be assessed with NMR [20, 27], thereby evaluating the process of cardiac energy metabolism. As a non-invasive technique, NMR can be performed repeatedly as a continuous monitoring in clinical practice and therefore is important. In the setting of DM, it may aid especially in the detection and diagnosis of early stage DM [28].

Our study further confirmed that cardiac energy metabolism was impaired in DM. Cardiac index significantly increased while cardiac ATP and pCr concentration remarkably decreased. Triptolide probably inhibited cardiac remodeling through the immune and inflammatory suppression [12–14]. However, whether the improvement of cardiac energy metabolism was achieved via the same mechanism remains largely unclear. On one hand, it is possible that the inhibition of cardiac remodeling could improve left ventricular geometry and systolic function. This will increase cellular blood and oxygen supply which eventually improve the process of cardiac energy metabolism [3, 6]. On the other hand, our study revealed that MAPK signaling pathway may be involved in the process. MAPK mRNA and protein expression which significantly increased in Triptolide treated group suggested that Triptolide probably improve cardiac energy metabolism at least partly through MAPK signaling pathway. MAPK signaling pathway is known as the most important pathway that interacts with mitochondria in the production of ATP [16]. Mitochondria have been known as the main factory for ATP production. Interestingly, through electron microscopic analysis, we observed an increasing number of mitochondria following Triptolide treatment.

Despite significant improvement of LVEF, fractional shortening (FS) in triptolide-treatment diabetic groups only showed the upward trend compared with the untreated diabetic group. The difference did not reach statistical significance. This may due to sampling error as FS value itself is lower than EF value. We believe that by increasing sample size, the difference will reach the statistical significance.

Cardiac index and LVEDD were negatively correlated with cardiac ATP and pCr concentrations, suggesting that the cardiomyocyte high-energy phosphate bond decreased after cardiac remodeling induced by DM. The decrease of intra-mitochondrial energy production characterized the process and Triptolide can partially reverse the process. This further affirmed the potential value of Triptolide treatment in diabetic cardiomyopathy.

## Conclusion

In the present study, we show that the abnormalities of cardiac energy metabolism in DCM rats could be improved partially by triptolide therapy. The improvement of cardiac energy metabolism following triptolide is at least partly through the upregulation of MAPK signaling transduction. ^31^P NMR spectroscopy enables the assessment of cardiac high-energy phosphates metabolism and therefore is able to evaluate the cardiac energy metabolism.
